# Is urotherapy alone as effective as a combination of urotherapy and biofeedback in children with dysfunctional voiding?

**DOI:** 10.1590/S1677-5538.IBJU.2018.0194

**Published:** 2018

**Authors:** Adem Altunkol, Deniz Abat, Nevzat Can Sener, Mehmet Gulum, Halil Ciftci, Murat Savas, Ercan Yeni

**Affiliations:** 1 Department of Urology, Adana City Teaching and Research Hospital, University of Healthy Sciences, Adana, Turkey;; 2 Department of Urology, Ministry of Health, Iskenderun State Hospital, Hatay, Turkey;; 3 Department of Urology, Ankara Keçiören Teaching and Research Hospital, University of Healthy Sciences, Ankara, Turkey;; 4 Department of Urology, Faculty of Medicine, Harran University, Şanliurfa, Turkey;; 5 Department of Urology, Antalya Teaching and Research Hospital, University of Healthy Sciences, Antalya, Turkey;; 6 Department of Urology, Ankara Numune Teaching and Research Hospital, University of Healthy Sciences, Ankara, Turkey

**Keywords:** Biofeedback, Psychology, Child, Urinary Tract Infections

## Abstract

**Objective:**

To compare standard urotherapy with a combination of urotherapy and biofeedback sessions and to determine the changes that these therapies promote in children with dysfunctional voiding.

**Patients and Methods:**

The data of 45 patients who participated in the study from January 2010 to March 2013 were evaluated. All patients underwent urinary system ultrasonography to determine post-void residual urine volumes and urinary system anomalies. All patients were diagnosed using uroflowmetry - electromyography (EMG). The flow pattern, maximum flow rate, and urethral sphincter activity were evaluated in all patients using uroflowmetry - EMG. Each patient underwent standard urotherapy, and the results were recorded. Subsequently, biofeedback sessions were added for all patients, and the changes in the results were recorded and statistically compared.

**Results:**

A total of forty - five patients were included, of which 34 were female and 11 were male and the average age of the patients was 8.4 ± 2.44 years (range: 5 - 15 years). After the standard urotherapy plus biofeedback sessions, the post-void residual urine volumes, incontinence rates and infection rates of patients were significantly lower than those with the standard urotherapy (p < 0.05). A statistically significant improvement in voiding symptoms was observed after the addition of biofeedback sessions to the standard urotherapy compared with the standard urotherapy alone (p < 0.05).

**Conclusions:**

Our study showed that a combination of urotherapy and biofeedback was more effective in decreasing urinary incontinence rates, infection rates and post - void residual urine volumes in children with dysfunctional voiding than standard urotherapy alone, and it also showed that this combination therapy corrected voiding patterns significantly and objectively.

## INTRODUCTION

Dysfunctional voiding (DV) is a dysmotility disorder that is unrelated to any obvious neurological or structural disease and is associated with dyssnergic sphincter activity. The term DV cannot be used unless a staccato pattern is observed in uroflowmetry measurements. DV presents as voiding difficulty in adults. However, it presents as nocturnal enuresis, urinary incontinence and / or recurrent urinary tract infections in children, and presumably, it may cause deformation of the upper urinary tract due to renal scarring (1). In children, there is a gradual increase in electromyography (EMG) activity due to increased pressure in the pelvic floor and urethral sphincters during the bladder filling phase, and the absence of EMG activity during voiding is defined as a normal EMG pattern by the International Children's Continence Society (ICCS). A normal voiding pattern is characterized by an uroflowmetry pattern in the form of a bell curve with a high peak flow, absence of a cut - off, no or minimal EMG activity during voiding, and a post - void residual (PVR) urine volume of less than 20 mL or 10% of bladder capacity in repeated measurements for children 4-6 years of age or a PVR urine volume of less than 10 mL or 6% of bladder capacity in repeated measurements for children 7-12 years of age (1, 2). Despite the lack of definitive information on DV, its incidence is increasing and varies between 2% and 25% in children (3, 4).

Treatment methods for DV include cognitive, behavioral, physical, and pharmacological therapies. Standard urotherapy is a noninvasive and nonpharmacological treatment and includes informing the child about the lower urinary system function and the child's differences from the norms, normal voiding habits, and voiding posture. It also provides lifestyle recommendations and training on retention maneuvers, fluid intake regulation, incapacity avoidance, maintenance of a bladder diary and voiding frequency schedule, and the importance of support and encouragement by the family (1). Biofeedback is also a nonpharmacological and non - surgical treatment. Biofeedback is defined as bladder training and rehabilitation (2, 5, 6). Maizels et al. first described biofeedback in 1979, and McKenna et al. first described animated biofeedback in 1999 (7). The biofeedback technique aims to ensure that the pelvic floor muscles, which play an important role in voiding control, are used correctly and effectively (1).

Our study aimed to compare the standard urotherapy and standard urotherapy combined with biofeedback regarding changes in symptoms in children with DV.

## PATIENTS AND METHODS

The study was approved by University of Health Sciences Adana City Teaching and Research Hospital Local Ethical Committee and informed consent was obtained from the parent's / caretakers of all the patients. After receiving the approval of the ethics committee, the data of 45 patients whose social - cultural level is low were retrospectively collected from two different centers in Turkey. The data of patients who participated in the study from January 2010 to March 2013 were evaluated. The average follow-up period of the patients was 24.6 ± 9.18 (range: 6 - 37) months. The symptoms of the patients were recorded as a percentage and were based on information from their parents or caregivers. A neurological examination of all patients was performed by a pediatrician. The urine analysis, plasma urea and creatinine values and urine culture results were evaluated in all patients. All patients underwent urinary system ultrasonography (USG) to determine PVR urine volumes and urinary system anomalies. All patients were diagnosed using uroflowmetry - EMG. The flow pattern, maximum flow rate and urethral sphincter activity were evaluated using uroflowmetry - EMG. To establish diagnosis, we performed two spontaneous flows. Urinary diaries were obtained from patients who had at least two days of attendance. Bladder diaries were obtained from patients before and after urotherapy and following biofeedback sessions.

Patients who were diagnosed with voiding dysfunction by uroflowmetry - EMG and older than five years of age were included in the study. Patients with a neurogenic bladder, anatomical malformations associated with incontinence, or a vesicoureteral reflux (VUR) history and patients who were less than 5 years old, were reluctant to participate in behavioral therapy, were uncooperative, or had a previous urologic operation history were excluded from the study. In our clinic, children who are diagnosed with voiding dysfunction are first treated with urotherapy. Routinely, biofeedback is applied to those who are not benefiting from urotherapy treatment. Forty - five patients were first treated with standard urotherapy. For standard urotherapy, cognitive and behavioral training was provided, which included training on appropriate toilet postures, timed voiding (every two hours), and fluid intake, namely, two cups of regular fluid intake per meal and a glass of fluid intake between meals. Additionally, a fiber - rich diet was recommended to the patients with constipation. The patients with continuing constipation, despite recommendations, received lactulose.

The changes in symptoms from before to after treatment were evaluated and recorded. The same patients who underwent urotherapy were additionally treated with biofeedback. The patients were treated with 6 to 10 sessions of biofeedback, and their symptoms were evaluated and recorded post - treatment. The biofeedback system is a video game that makes patients move objects on the screen and allows them to learn how to flex their pelvic floor muscles. This system was explained in detail to each patient and his or her close relative. Patients were told to come to the lab with a full bladder. EMG electrodes were placed on both sides of the perineum at the 3 and 9 o'clock positions, and other electrodes were placed on the rectus muscle to measure abdominal muscle activity. During the first session, the patient was told to contract and relax to move the objects and was asked to stay as relaxed as possible. When the children understood this concept, home exercises were given to help them relax during voiding as much as possible. The results of this study were classified according to the updated ICSS as follows: no response (0 - 49% reduction in symptoms), partial response (50 - 99% reduction in symptoms), and complete response (100% loss of symptoms). Objective improvement was defined as a decrease in EMG activity during voiding, a normal voiding curve on uroflowmetry, and a PVR urine volume of < 20 mL (8). All patients were evaluated with uroflowmetry - EMG before treatment, after urotherapy and at the end of the biofeedback sessions. All biofeedback session consisted of a single successful flow. After a successful trial with biofeedback sessions, a single ‘normal’ flow made the final assessment. The responses of the patients were compared between these three time points.

### Statistical analyses

Statistical analyses were performed using the Statistical Package for Social Sciences version 20.0 software (SPSS Inc, Chicago, Illinois, USA). All groups were compared using Paired Samples T test for continuous variables, and the Chi Square or Fisher exact test for categorical variables. Two - tailed p values < 0.05 was considered as statistically significant.

## RESULTS

A total of forty - five patients were included, of which 34 were female and 11 were male, and the average age of the patients was 8.4 ± 2.44 (range: 5 - 15) years. The average initial PVR urine volume was 68.4 ± 17.76 (range: 40 - 130) mL. The average PVR urine volume after urotherapy was 55.29 ± 15.06 (range: 15 - 88) mL, and the average PVR urine volume after the urotherapy plus biofeedback combination was 28.42 ± 17.25 (range: 5 - 88) mL. The PVR urine volumes were significantly decreased following the combined treatment compared with standard urotherapy alone (p = 0.000). Initially, 16 of the 45 (35.55%) patients had urinary system infections. Recurrent urinary tract infections were detected in 10 of the 45 patients after standard urotherapy (22.2%) and in only 2 of the 45 patients (4.44%) after the urotherapy plus biofeedback combination. The rate of recurrent urinary infection was found to be significantly lower in the combination therapy group (p = 0.002). Initially, 29 of the 45 patients had (64.44%) urinary incontinence. Following standard urotherapy, 18 (40%) patients had continuing incontinence, while only 4 (8.88%) patients had continuing urinary incontinence after the urotherapy plus biofeedback combination therapy, which was a statistically significant difference (p = 0.01). These results are shown in Table-1.

**Table 1:** Characteristics and statistical comparisons of the treatment protocols applied to the patients.

	Initial	After Standard Urotherapy (n=45)	After the Urotherapy Plus Biofeedback Combination Therapy (n=45)	P Value
**Urinary Tract Infection** **(Number/percent)**	16 (35.55%)	10 (22.2 %)	2 (4.44%)	0.002
**Urinary Incontinence** **(Number/percent)**	29 (64.44%)	18 (40 %)	4 (8.88%)	0.01
**Mean pvR Urine volume** **(Mean±SD)**	68.4±17.76 (40-130)	55.29±15.06 (15-88)	28.42±17.25 (5-88)	* 0.000 ** 0.000 *** 0.000

P< 0.05 was considered statistically significant.

PVR Urine Volume

* Initial—After Standard Urotherapy

** Initial- After the urotherapy plus biofeedback combination therapy

*** After Standard Urotherapy - After the urotherapy plus biofeedback combination therapy

**Figure 1:**
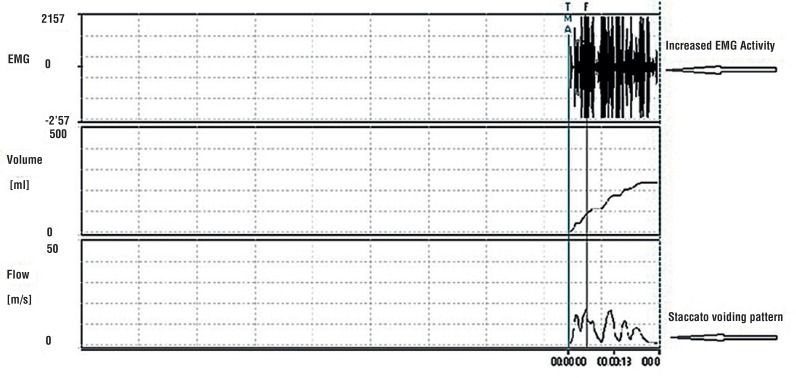
Uroflowmetry-EMG image from a 7-year-old girl before therapy.

**Figure 2:**
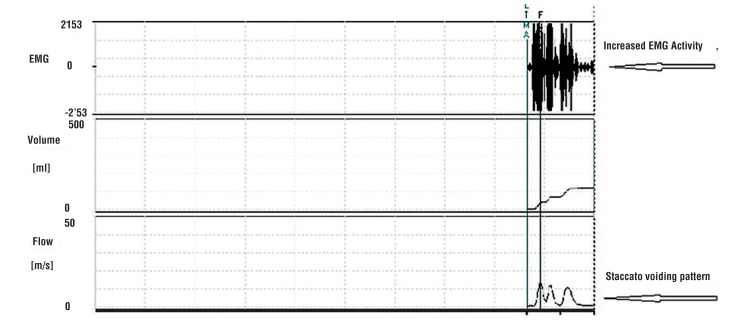
The uroflowmetry - EMG image after standard urotherapy.

**Figure 3:**
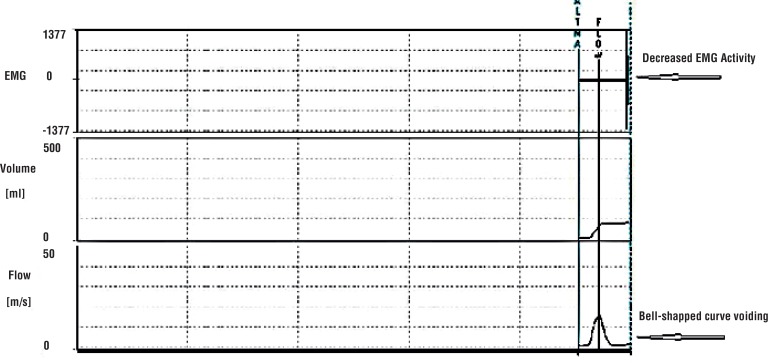
The uroflowmetry - EMG image after standard urotherapy plus biofeedback sessions. (All images were obtained from the same patient.)

All patients had abnormal voiding patterns when they were admitted to the clinic. After standard urotherapy, 27 of the 45 patients (60%) had a voiding pattern that became bell curve - shaped, while 18 (40%) patients still had a staccato pattern. Thirty - eight (84.4%) patients exhibited improvement in the voiding pattern following the urotherapy plus biofeedback combination treatment, while 7 (15.5%) had no improvement. The changes in voiding patterns measured by uroflowmetry of one patient are shown in Figures 1–3 as EMG images. There was no response to standard urotherapy in 20 patients (44.4%), while the combination of urotherapy plus biofeedback left 4 (8.8%) patients with consistent symptoms. All of those 4 patients had no response to standard urotherapy. A partial response was obtained in 25 (55.5%) patients after standard urotherapy, and a partial response was obtained in 24 (53.3%) patients after the urotherapy plus biofeedback combination. All 20 patients with no response to urotherapy showed partial recovery from urotherapy and biofeedback combination (20 / 24). Complete recovery was obtained in 17 (37.7%) patients after the urotherapy plus biofeedback combination, while no patients showed complete recovery (0%) after standard urotherapy. Patients with complete recovery were symptom free after 6.82 ± 0.88 (6–9) sessions of biofeedback. Patients without complete recovery received 10 biofeedback sessions. A statistically significant improvement was seen in the symptoms of patients following the urotherapy plus biofeedback combination (p = 0.004, Table-2).

**Table 2:** Changes in symptoms after standard urotherapy and after the urotherapy plus biofeedback combination therapy.

	Standard Urotherapy (n:45)	Urotherapy Plus Biofeedback Combination Therapy (n:45)	P Value
No Response	20 (44.4%)	4 (8.8%)	
Partial Response	25 (55.5%)	24 (53.3%)	0.004*
Complete Response	0 (0%)	17 (37.7%)	

## DISCUSSION

DV is a health problem that shows an increasing frequency among children. Daytime urinary incontinence is encountered in 3.8% of boys and 6% of girls who are seven years of age. This condition has significant effects on the quality of life of the child and his or her family. The primary importance of DV is associated with the urinary tract infections and/or VUR. For this reason, receiving a DV diagnosis and undergoing appropriate treatment are important (9, 10). In the diagnosis of DV, there is a preference for noninvasive tests rather than invasive urodynamic examinations. Cystometry is mostly used in selected cases, and advanced diagnostic tools such as videourodynamics and magnetic resonance imaging are used in complicated and / or unresponsive patients. Uroflowmetry - EMG, a noninvasive test that can diagnose lower urinary tract dysfunction, provides important information about the response to treatment.

Irkilata and colleagues reported that in the study of children with dysfunctional voiding, uroflowmetry - EMG, as a noninvasive method, should be performed first for diagnosis and in the evaluation of treatment response. However, it has also been suggested that to minimize the influence of the outside environment on the patient and increase the reliability of the test, appropriate environmental conditions should be created (11). Additionally, in another study, the authors reported that the ratio of the bladder wall thickness to the empty bladder wall thickness obtained from ultrasound, which is used to assess lower urinary tract symptoms in pediatric patients with dysfunctional voiding, could be used as a noninvasive, inexpensive, simple and rapid tool, but this still needs to be combined with other noninvasive diagnostic tests (9). The clinical symptoms related to dysfunctional voiding are nonspecific, and utilization of uroflowmetry - EMG could improve diagnosis and treatment (12).

In our study, we also used uroflowmetry -EMG. This is a convenient diagnostic tool, which can provide information on the diagnosis, treatment and the follow-up of the patients at the same time.

Standard urotherapy and / or biofeedback therapy have been used among the dysfunctional voiding treatment modalities (1, 2, 5, 6). Standard urotherapy increases pelvic floor muscle function and the awareness of relaxation in children with DV; it also promotes the relaxation of the lower abdominal muscles (transversus and obliquus internus abdominis) (13).

Children with lower urinary dysfunction characterized by incontinence were treated with urotherapy by Mulders et al. They provided advice on situations such as toilet training, relaxation of the pelvic floor muscles, fluid intake, voiding scale and defecation regulation. They reported that more than 78% of the children were successful in preventing urinary incontinence, correcting voiding pattern, and decreasing voiding frequency and PVR urine volume (14). Additionally, studies have shown that urotherapy reduces the need for surgery in patients with VUR and decreases constipation and the number of urinary tract infections (15). In addition, several authors reported that in many studies without biofeedback, successful results were obtained with standard urotherapy (14, 16, 17).

In our study, after standard urotherapy, we observed reduced urinary tract infection to a percentage of 37.5%, incontinence to a percentage of 37.9% and the PVR volumes to a percentage of 19.1%. We observed that the voiding pattern showed a bell - shaped curve in 60% percent of the patients, and 40% percent of the patients continued to have the staccato pattern after the standard urotherapy. We concluded that the reason for the failure of standard urotherapy may be due to the low socio - cultural status and low education level of the parents and caretakers of the patients. The parent's belief that standard urotherapy is not an effective treatment method and the possibility of nonconformity to the treatment may have negative effects on our treatment results. Standard urotherapy is a behavioral treatment modality, and it is applied at home. Thus, this treatment is achieved in a joint operation by the doctors, patients and the parents, and it requires exact compliance to the doctor's recommendations. It is also very important that the parents and caretakers are educated about this subject. The other treatment modality is biofeedback therapy, and it is applied in hospital conditions. It is important to create an environment that has little external stimuli, that the patient can easily tolerate and that enables the patient to feel relaxed to obtain valuable information to facilitate treatment progression. It has also been reported that biofeedback performed with an animated program that keeps the attention of children and their parents improved treatment attendance and enhanced treatment applicability, thus enhancing the success of the treatment, under the supervision of an expert in the clinic (18). The animated program used in the biofeedback session is a game. Therefore, it reduces the treatment anxiety of the children and provides continuity of treatment by rewarding the children with prizes. In some studies, the authors suggested that the therapy should be continued if EMG activity is observed during the voiding phase period, without session limitations (2, 19, 20).

In this study, we provided 6 to 10 biofeedback treatment sessions to the children. These sessions were ended after an improvement in the uroflowmetry - EMG traces. We believe the differences in number of sessions was due to the patient's understanding of urotherapy as well as that of their parents and how well they have cooperated with their physician. We believe the number of sessions will be lower for the patients with improved cooperation.

Peco - Antic et al. reported that the combined standard urotherapy plus biofeedback in girls with lower urinary system dysfunction reduced urinary tract infections and constipation frequency and improved urinary incontinence (6). Similarly, another study by Kibar and colleagues reported a significant improvement in outcomes in children with DV using combined urotherapy plus biofeedback compared with standard urotherapy (2). Another study reported that symptomatic healing was observed in 50% of children with urotherapy - resistant DV who were treated with biofeedback (21). In recent years, biofeedback therapy has been increasingly used in children with DV. With this method, urinary incontinence, urinary tract infection, PVR urine volumes, and constipation were improved (22). Treatment success rates are assessed based on symptoms of urinary tract infections, voiding patterns, PVR urine volumes, and incontinence changes in patients (14). Recurrent urinary tract infection is the most serious cause of morbidity in children with dysfunctional voiding (23). The causes of infections in DV include incomplete bladder emptying and increased residual urine volumes (24).

In our study, we initially found infection in 16 (35.55%) of our patients; however, after urotherapy and the combination of urotherapy and biofeedback, the infection persisted in 10 (22.2%) and 2 (4.44%) patients, respectively, indicating that several patients exhibited improved bladder function after the combination therapy. This result suggests that bladder function is improved with combination therapy in a greater number of children with DV. However, we found that the decreases in PVR urine volume from baseline to the post - urotherapy time point, from baseline to the post - combination therapy time point, and from the post - urotherapy time point to the post - combination therapy time point were significant.

Although this decrease was statistically significant, PVR urine volumes after urotherapy in many patients were not < 20 mL; therefore, they did not meet the ICCS criteria (8). In this case, the results indicate the possibility for urinary tract infections and serious morbidity only after urotherapy, as the bladder of these patients did not show the appropriate outpouring according to the ICCS criteria.

We were unable to obtain a complete response of symptoms using only urotherapy. However, we obtained a statistically significant response level with the combination of urotherapy plus biofeedback sessions, and this combination therapy improved the voiding pattern that was shown by uroflowmetry - EMG. We compared our results according to the literature with respect to symptomatic responses, incontinence, urinary tract infections, and voiding patterns.

We did not obtain any complete responses from the standard urotherapy; we only obtained partial responses. However, when we added biofeedback sessions to urotherapy, 4 (8.8%) patients did not show any response, 24 (53.3%) patients showed a partial response, and 17 (37.7%) patients exhibited a complete response, and these results were statistically significant. We also noted that the voiding patterns of 60% of the patients were bell curve - shaped when we examined the results of uroflowmetry - EMG. Thus, the addition of biofeedback to urotherapy resulted in a statistically significant improvement in DV in accordance with the objective improvement criteria determined by the ICCS.

The low number of patients, the fact that the data were collected from two centers, and the retrospective nature of the study were limitations of the present study. Also, the therapy provided for the patients were supervised by not the physicians but either parents or caregivers of children. In this socioeconomic environment, this could be attributed as a limitation.

## CONCLUSIONS

We observed that the combination of urotherapy plus biofeedback in children with dysfunctional voiding reduced urinary incontinence rates, infection rates and PVR urine volumes and corrected voiding patterns at an objective level; the combination was better than only standard urotherapy. Standard urotherapy is a training modality that requires patient compliance and exact compliance with recommendations of specialists. We hypothesized that the low success rate of our standard urotherapy after adding biofeedback was due to the effect of low socio - cultural level of the region where the treatments were performed. However, prospective studies involving large numbers of patients are needed to verify this conclusion.

### Ethical approval

The study was approved by University of Health Sciences Adana City Teaching and Research Hospital Local Ethical Committee.

All procedures performed in studies involving human participants were in accordance with the ethical standards of the institutional and/or national research committee and with the 1964 Helsinki declaration and its later amendments or comparable ethical standards.

Informed consent was obtained from the parent's / caretakers of all the patients.
